# Spatial variation and determinants of delayed breastfeeding initiation in Ethiopia: spatial and multilevel analysis of recent evidence from EDHS 2019

**DOI:** 10.1186/s13006-024-00616-1

**Published:** 2024-02-07

**Authors:** Ribka Nigatu Haile, Biruk Beletew Abate, Tegene Atamenta Kitaw

**Affiliations:** Department of Nursing, College of Health Science, Woldia University, Woldia, Ethiopia

**Keywords:** Spatial, Multilevel analysis, Delayed breastfeeding initiation, Determinants

## Abstract

**Background:**

Despite the World Health Organization’s firm recommendation to start breastfeeding during the first hour after delivery, nearly 54% of children in low- and middle-income countries are unable to initiate breastfeeding within the recommended time frame. Understanding the initiation of breastfeeding is essential for optimal child health and maternal well-being.

**Methods:**

This study was conducted using the recent Ethiopian Demographic and Health Survey (EDHS) data (2019) on a weighted sample of 1982 Ethiopian mothers of children aged under 24 months. The data extraction was conducted between August 1 and 30, 2023. Delayed’ initiation of breastfeeding is defined as failure to initiate breastfeeding within one hour after birth. STATA version 17 was used for non-spatial analysis. ArcGIS Pro and Sat Scan version 9.6 were used to map the visual presentation of delayed breastfeeding initiation. Global Moran’s I was computed to determine whether delayed breastfeeding initiation is randomly distributed, clustered, or dispersed. Getis-Ord Gi* Spatial Statistics was done to identify significant spatial clusters of cold and hot spot areas. Multilevel mixed-effect logistic regression analysis was computed to identify determinants of delayed breastfeeding initiation.

**Results:**

The prevalence of delayed breastfeeding initiation is 26.4% (95% CI 24.4, 28.3). Significant clustering of delayed initiation of breastfeeding practice was found in the Somali region. Less clustering was identified in Northern Amhara, Addis Ababa and Dire Dawa. Being a young mother (15–24 years) (AOR 1.66; 95% CI 1.06, 2.62), no antenatal care (AOR 1.45; 95% CI 1.04, 2.02), cesarean section (AOR 4.79; 95% CI 3.19, 7.21) and home birth (AOR 1.53; 95% CI 1.14, 2.06) were found to be determinants of delayed initiation of breastfeeding.

**Conclusions:**

In Ethiopia, delayed breastfeeding initiation is distributed non-randomly. Significant hotspot areas were identified in the eastern part of Ethiopia. Thus, deploying additional resources in high hotspot regions is recommended. Programs should focus on promoting health facility birth and increasing antenatal care visits. Further emphasis should be considered on supporting young mothers and those giving birth via cesarean section to improve timely breastfeeding initiation.

**Supplementary Information:**

The online version contains supplementary material available at 10.1186/s13006-024-00616-1.

## Background

Despite significant recommendations from the United Nations International Children’s Emergency Fund (UNICEF) and the World Health Organization (WHO) to start breastfeeding during the first hour after delivery, many neonates are not breastfed within the recommended time [1]. Worldwide, three out of every five neonates had delayed breastfeeding initiation. In 2017 alone, approximately 78 million newborn babies did not start breastfeeding within one hour of birth [2]. In low- and middle-income regions, 53.8% is the prevalence of late breastfeeding initiation. It ranges from 15.0% in Burundi to 83.4% in Guinea [3]. In Ethiopia, nearly 39% of children do not start breastfeeding within the recommended time [4].

Late breastfeeding initiation is associated with severe sepsis, thereby increasing neonatal death [3]. It is also linked with prelacteal feeding practices, which can increase the likelihood of hospitalization, diarrhea, and lower respiratory tract infections [5]. On-time breastfeeding initiation is also associated with a decreased chance of postpartum hemorrhage [6]. Furthermore, timely breastfeeding initiation increases maternal-infant bonding, which significantly contributes to early child development [7].

Recent studies urge that the risk of neonatal mortality rises by 33% among neonates who start breastfeeding beyond one hour after birth [8].

Factors affecting delayed breastfeeding initiation were identified in different studies. Such as being a young-aged mother [9], rural resident [10], unmarried mother [11], place of birth [12], mothers educational level [13], mode of delivery [14], media exposure [15], mother body mass index [16], parity [17], frequency of antenatal care [18], poor wealth index [19] and births attended by skilled health personnel [20].

By 2030, The Sustainable Development Goals (SDGs) aim to decrease newborn and under-five mortality to as low as 12 and 25 per 1,000 live births, respectively [21]. In achieving the above plan, initiating breastfeeding within one hour of birth and exclusively breastfeeding for the first six months of a child’s life play a prominent role. Besides, the Ethiopia Health Sector Transformation Plan (HSTP) emphasizes achieving optimal breastfeeding practices to reduce under-five mortalities from 59 to 36 per 1000 live births [22].

Despite the enormous progress made previously and the known health benefits of optimal breastfeeding practice, ending delayed breastfeeding initiation in developing countries like Ethiopia has become a significant challenge. Most studies regarding breastfeeding practice in Ethiopia are limited to specific districts/areas. Recent data regarding breastfeeding practice is crucial for public health practitioners and policymakers in supporting efforts towards strengthening child nutrition programs, and ending all types of malnutrition in the late life of children.

The study was carried out using recent data (2019) from the Ethiopia Demographic and Health Survey (EDHS), which is crucial in providing up-to-date information on national improvements regarding breastfeeding practice. Understanding factors determining delayed initiation of breastfeeding practice is essential in designing strategies for improving child nutrition. In addition, identifying hotspots or areas where delayed breastfeeding initiation is higher than the national average is critical to accelerate intervention toward breastfeeding practice. Thus, the study sought to determine the spatial variation and determinants of delayed breastfeeding initiation in Ethiopia.

## Methods

### Study setting, study period and data source

According to forecasts from trading economics and data from recent census figures, the total population of Ethiopia was 115.0 million by 2020 [23]. The EDHS 2019 final report includes inclusive data at the country level from the nine regional states and two municipal administrations. The administrative levels were divided into zones, woreda, and so forth. Spatial and multilevel analysis were conducted on children less than 24 months. Women between the ages of 15 and 49 and children in selected households across the country were the target groups. The EDHS collects pertinent information mainly regarding maternity health care utilization, marriage and sexual behavior, child feeding practice, children and women’s dietary condition, and children’s and adult mortality. Data collection was carried out from March to June 2019 [24].

### Data extraction and population

First, the project proposal was sent to the Demographic and Health Surveys (DHS) Program. After a detailed review process, the DHS program accepted the proposal and granted access with an approval letter to use the survey datasets. Data extraction was done to select mothers of children aged less than twenty-four months. The data extraction was conducted between August 1 and 30, 2023. All Ethiopian mother of children aged less than twenty-four months were the source population, whereas all Ethiopian mother of children aged less than twenty-four months in the selected enumeration area were the study population.

### Data quality control and data collection

To ensure the integrity and reliability of data gathered for this research, the DHS quality control team employed robust data quality control mechanisms. During data collection, data collection instruments and procedures were meticulously reviewed to minimize errors and inconsistencies. As per the EDHS 2019 report, Interviewers were trained on the DHS questionnaires and data collection procedures. Data collection spanned from March to June 2019. Following the selection of a representative sample of households across nine regions and two administrations, Interviewers visited selected households to conduct interviews with women of reproductive age. The interviews were typically conducted in the respondent’s home. For this study, data profiling techniques were also employed to identify data anomalies, outliers, and missing values. Additionally, recourse methodology was utilized throughout the study.

### Sampling methods

**Fig. 1:**
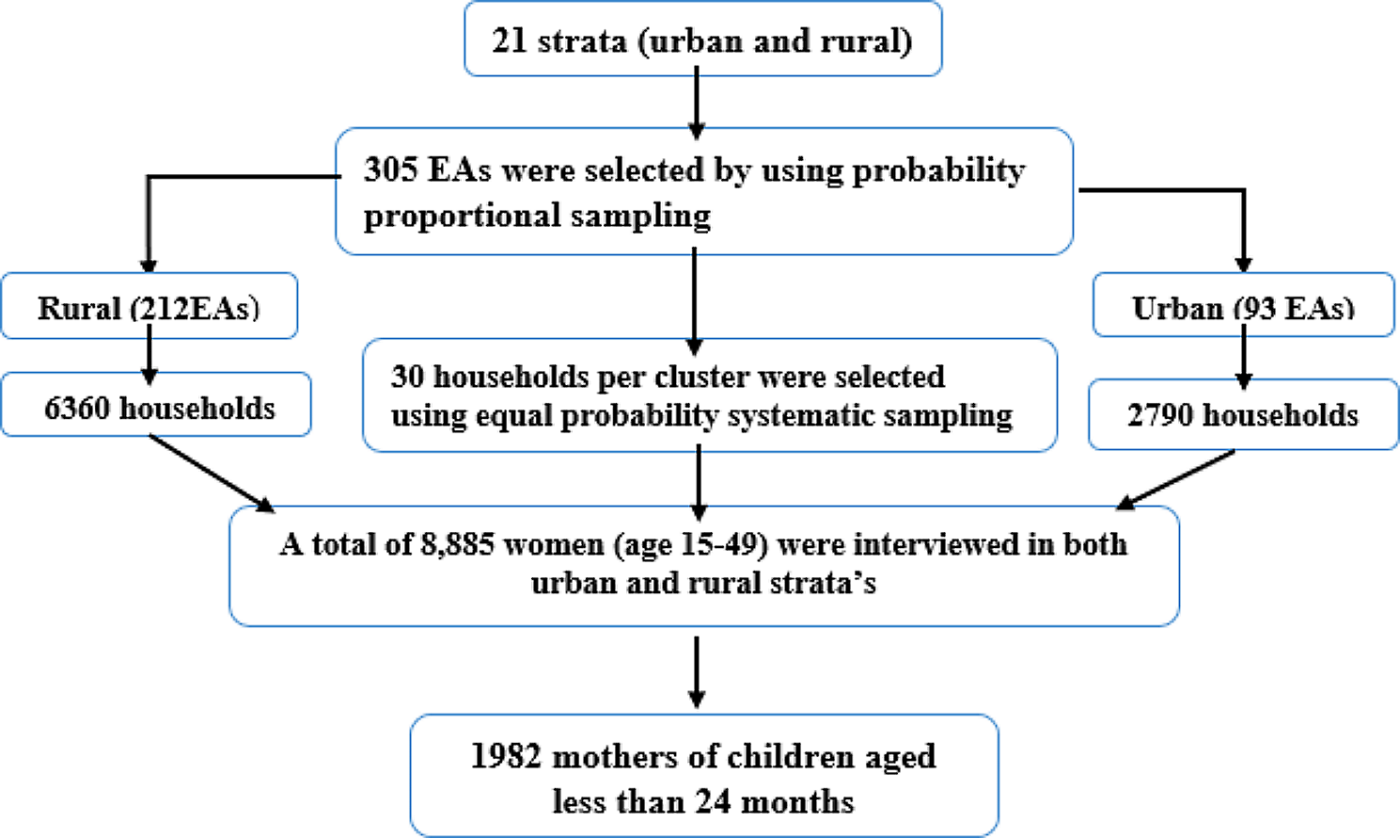
Schematic representation of the sampling procedures in the study of spatial variation and determinants of delayed breastfeeding initiation in Ethiopia, EDHS, 2019 N.B EAs = Enumeration Areas

The EDHS 2019 sample was stratified and selected in two levels. Twenty one sampling strata were produced after stratifying each region into urban and rural areas. Using probability proportion, 305 enumeration areas (93 from urban and 212 from rural) were selected in the first stage. Newly formed household listing was used in the second stage to choose a set number of 30 households per cluster with an equal probability of systematic selection. Sample allocation was done to verify that survey precision was equivalent across regions. Thirty five enumeration areas were selected from the three largest regions. Twenty five enumeration areas were selected from eight regions (including two city administrations). The complete sampling procedure is available in the EDHS 2019 final report. In the current study, a total of 1982 weighted mothers of children under twenty-four months participated. The spotlight sampling technique for the present study is shown in the Fig. 1.

### Study variables

The dependent variable is delayed breastfeeding initiation. This study considered different independent variables to identify determinants of delayed breastfeeding initiation. (Table 1). The community poverty level is constructed using individual-level factors at the cluster (community) level, thereby classified as lower or higher by using median value as a cut-off point if the distribution is not normal. Furthermore, the distribution was checked by using a histogram.

**Table 1:** List of independent variables for the assessment of Spatial variation and determinants of delayed breastfeeding initiation in Ethiopia

Variable	Descriptions (classification)
Mother age	15–24, 25–34, 35–49
Residence status	Urban or Rural
Religion	Orthodox, Protestant, Muslim, Other
Region	Larger central: Tigray, Amhara, Oromia, SNNPR Small peripherals: Benishangul, Gambela, Afar, Somali Metropolis: Harari, Addis Ababa, Dire Dawa.
Mother educational status	No education, Primary, Secondary or higher
Wealth index	Poor, Middle, and Rich
Marital status	Not married or married
Antenatal	No ANC visit, 1–3 or 4 and above
Place of delivery	Home, or health institution
Mode of delivery	SVD or Cesarean section
Type of birth	Single or twin and above
Sex of child	Male or Female
Parity	1, 2–3, and 4 and above
Community poverty level	Higher or lower

### Definitions

Delayed initiation of breastfeeding is defined as failure to initiate breastfeeding within one hour after birth as WHO recommendation [25]. The outcome variable is dichotomized as “1” for delayed initiation and “0” for timely initiation.

### Data processing and analysis

Data was extracted from the EDHS 2019 individual record folder using STATA version 17. The data was sorted and listed to identify any missing values. Frequency and percentage were computed for descriptive statistics. Date weighting, cleaning, editing, and recording were carried out. ArcGIS Pro and Sat Scan version 9.6 were used for mapping the visual presentation of delayed breastfeeding initiation at the regional and district levels. STATA version 17 was used for descriptive and non-spatial analysis.

#### Spatial autocorrelation analysis

Spatial autocorrelation with Global Moran’s I was computed to determine whether delayed breastfeeding imitation is randomly distributed, clustered, or dispersed. Global Moran’s I value near “0” indicates delayed breastfeeding is randomly distributed, near “–1” shows dispersed, and close to “+1” indicates clustered. Spatial autocorrelation is declared at a statistically significant Moran’s I *P* - value less than 0.05.

#### Hot spot analysis

Hot Spot Analysis was done using Getis-Ord Gi* Spatial Statistics to identify statistically significant spatial clusters of minimum value (cold spots) and maximum value (hot spots). *P* - values and Z-score were used to measure statistical significance, thereby deciding whether or not to reject the null hypothesis. The null hypothesis could not be rejected if the Z-score value is between − 1.96 and + 1.96 at a *P* - value greater than 0.05. The null hypothesis was rejected if the Z-score is not within the range at a *P* - value of significance. The possible cause of a statistically significant spatial pattern was explored further. In addition, a high Gi* statistical result shows a “hotspot,” and a low Gi* revealed a “cold spot.”

#### Spatial interpolation

Spatial interpolation was employed to estimate the value of unsampled areas from sampled data points. Empirical Bayesian Kriging (EBK) was used to account for the error made by estimating the semi variogram model.

#### Multilevel logistic regression analysis

Multilevel mixed-effect logistic regression analysis was computed to identify significant determinants of delayed breastfeeding initiation. Multilevel modelling is a statistical model used to analyze data drawn from different levels.

Model one (null model): $\mathbf{logit}\left(\varvec{\pi }\varvec{i}\varvec{j}\right)=\varvec{\beta }\mathbf{\varnothing }\mathbf{\varnothing }+\varvec{u}\mathbf{\varnothing }\varvec{j}:$where: πij is the probability of the outcome (delayed breastfeeding initiation) for individual i in community j, β00 is the overall intercept and u0j is the random effect for community j.

Model two (Individual-Level Factor): $\text{l}\text{o}\text{g}\text{i}\text{t}\left(\pi ij\right)=\beta {\varnothing}0+\beta 1\times ij+u{\varnothing}j$: where: xij is the value of the individual-level factor for individual i in community j and β1 is the coefficient for the individual-level factor.

Model three (Community-Level Factor): $\mathbf{l}\mathbf{o}\mathbf{g}\mathbf{i}\mathbf{t}\left(\varvec{\pi }\varvec{i}\varvec{j}\right)=\varvec{\beta }00+\varvec{\beta }2\varvec{z}\mathbf{\varnothing }\varvec{j}+\varvec{u}\mathbf{\varnothing }\varvec{j}$: where: z0j is the value of the community-level factor for community j and β2 is the coefficient for the community-level factor.

Model four (Both Individual- and Community-Level Factors): $\mathbf{l}\mathbf{o}\mathbf{g}\mathbf{i}\mathbf{t}\left(\varvec{\pi }\varvec{i}\varvec{j}\right)=\varvec{\beta }\mathbf{\varnothing }\mathbf{\varnothing }+\varvec{\beta }1\times \varvec{i}\varvec{j}+\varvec{\beta }2\varvec{z}\mathbf{\varnothing }\varvec{j}+\varvec{u}0\varvec{j}$: where: xij is the value of the individual-level factor for individual i in community j, β1 is the coefficient for the individual-level factor, z0j is the value of the community-level factor for community j and β2 is the coefficient for the community-level factor [26].

A multilevel model was fitted because of the hierarchical nature of the EDHS data. Four models were considered. Model 1 considers only the dependent variable to explore the degree of cluster variation on delayed breastfeeding initiation. Model 2 and Model 3 contain individual-level factors and community-level factors, respectively. Model 4 is adjusted for both individual and community levels concurrently. Adjusted odds ratio (AOR) and respective 95% confidence interval were computed to identify significant determinants of delayed breastfeeding initiation. Variance inflation factor and tolerance value were used to check the exitances of multicollinearity between variables. A VIF above 4 or tolerance below 0.25 indicated multicollinearity might exist [27]. To estimate the variation between clusters, proportional change variance (PCV), intra-class correlation (ICC), and median odd ratio (MOR) were computed.

ICC shows the degree of heterogeneity of delayed breastfeeding initiation between clusters and calculated as:

$$ ICC=\frac{{\sigma }^{2}}{\left({\sigma }^{2}+{\sigma }_{b}^{2}\right)},$$

where σ^2^ represent community level variance, ${\sigma }_{b}^{2}$ indicates individual level variance [28].

$$ {\sigma }_{b}^{2}=\frac{{\pi }^{2}}{3}$$

MOR is the median variations of odds ratio between high-risk areas of delayed breastfeeding initiation and low risk during randomly picking out of clusters. It is calculated as:

$$ \text{M}\text{O}\text{R}={e}^{\left[\sqrt{\left(2*{V}_{A}\right)0.6745}\right]}={e}^{\left[0.95*\sqrt{{V}_{A}}\right]},$$

where VA represents the area level variance.

PCV measures the total variation in delayed breastfeeding initiation attributable to factors in successive models. It is computed as: $PCV=\frac{V\text{ null }-VA}{V\text{ null }}*100\text{\%}$, where V_null_ is variance in null model and V_A_ is variance in successive model.

## Results

### Sociodemographic characteristics of study participants

A total of 1982 weighted study participants were included to explore the spatial variation of delayed breastfeeding initiation.1487 (75.03%) of respondents resided in rural areas. Concerning the mother’s educational level, 956 (48.23%) respondents have no formal education. 950 (47.93%) of mothers were in poor wealth index level. (Table 2).

**Table 2:** Sociodemographic characteristics of study participants in Ethiopia EDHS 2019. (*n* = 1982)

Variable	Categories	Weighted frequency (%)	Breastfeeding initiation time	
			**Early**	**Delayed**
Sex of the child	Male	985 (49.7%)	724 (49.6%)	261 (49.9%)
	Female	997 (50.3%)	735 (50.4%)	262 (50.1%)
Age of the mother	15–24	637 (32.1%)	452 (31.0%)	193 (36.9%)
	25–34	1036 (52.3%)	760 (52.0%)	272 (52.0%)
	35–49	309 (15.6%)	247 (17.0%)	58 (11.1%)
Religion	Orthodox	605 (30.5%)	428 (29.3%)	177 (33.8%)
	Protestant	372 (18.8%)	281 (19.3%)	91 (17.4%)
	Muslim	969 (48.9%)	723 (49.6%)	246 (47.0%)
	Other	36 (1.8%)	27 (1.9%)	9 (1.7%)
Place of residence	Urban	495 (24.9%)	372 (25.5%)	123 (23.5%)
	Rural	1487 (75.0%)	1087 (74.5%)	400 (76.5%)
Educational status	No education	956 (48.2%)	705 (48.3%)	251 (48.0%)
	Primary education	697 (35.2%)	510 (35.0%)	187 (35.8%)
	Secondary education	197 (9.9%)	143 (9.8%)	54 (10.3%)
	Higher education	132 (6.7%)	101 (6.9%)	31 (5.9%)
Marital status	Married	1866 (94.2%)	1370 (93.9%)	496 (94.8%)
	Not married	116 (5.9%)	89 (6.1%)	27 (5.2%)
Wealth index level	Poor	950 (47.9%)	682 (46.7%)	268 (51.2%)
	Middle	280 (14.1%)	209 (14.3%)	71 (13.6%)
	Rich	752 (37.9%)	568 (38.9%)	184 (35.2%)
Media access (television)	Yes	393 (19.8%)	294 (20.1%)	99 (18.9%)
	No	1589 (80.2%)	1165 (79.9%)	424 (81.1%)
Media access (radio)	Yes	506 (25.5%)	379 (26.0%)	127 (24.3%)
	No	1476 (74.5%)	1080 (74.0%)	396 (75.7%)

### Maternal and reproductive characteristics

Nearly 25% of the participants had no antenatal care visit, and 42.8% of the mothers gave birth at home. Seven-point 1% of mothers delivered their baby through cesarean section. (Table 3). The prevalence of delayed breastfeeding initiation is 26.4% (95% CI 24.4, 28.3).

**Table 3:** Maternal and reproductive characteristics of mothers in Ethiopia, EDHS 2019. (*n* = 1982)

Variables	Categories	Weighted frequency (%)	Breastfeeding initiation time	
			**Early**	**Delayed**
ANC visit	No ANC visit	505 (25.5%)	339 (23.2%)	166 (31.7%)
	1–3 visit	649 (32.8%)	484 (33.2%)	165 (31.6%)
	4 and above visit	828 (41.8%)	636 (43.6%)	192 (36.7%)
Place of delivery	Home	849 (42.8%)	591 (40.5%)	258 (49.3%)
	Health institution	1133 (57.2%)	868 (59.5%)	265 (50.7%)
Mode of delivery	SVD	1842 (92.9%)	1389 (95.2%)	453 (86.6%)
	Cesarean section	140 (7.1%)	70 (4.8%)	70 (13.4%)
Twin or single birth	Single birth	1964 (99.1%)	1446 (99.1%)	518 (99.0%)
	Twin	18 (0.9%)	13 (0.9%)	5 (1.0%)
Parity	1	449 (22.7%)	306 (21%)	143 (27.3%)
	2–3	710 (35.8%)	524(36%)	186 (35.6%)
	4 and above	823 (41.5%)	629 (43.1%)	194 (37.1%)
Birth order	1–3	1159 (58.5%)	830 (56.9%)	329 (62.9%)
	4–6	564(28.5%)	429 (29.4%)	135 (25.8%)
	6 and above	259 (13.0%)	200 (13.7%)	59 (11.3%)

### Spatial and incremental autocorrelation

**Fig. 2:**
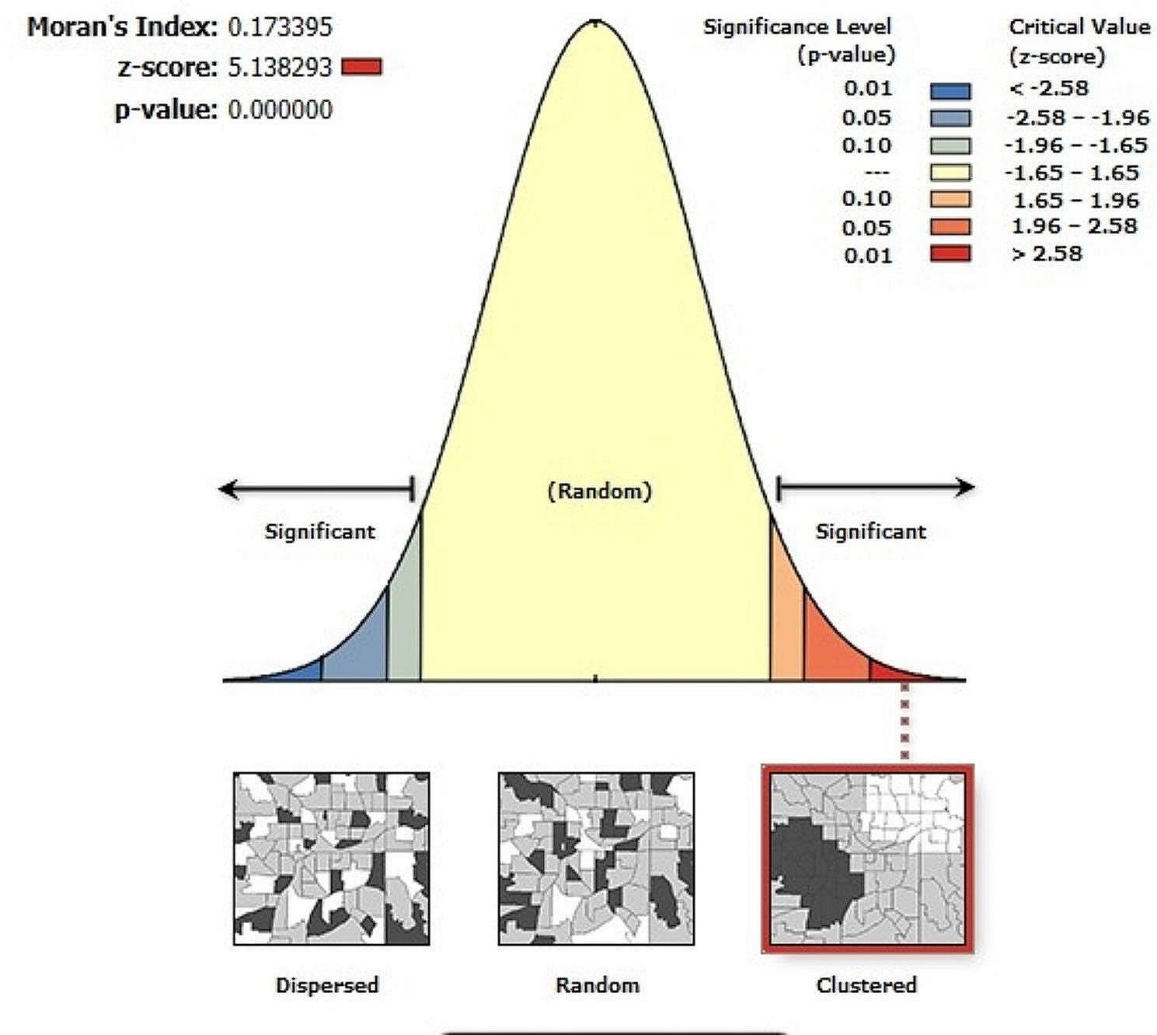
Spatial autocorrelation analysis of delayed breastfeeding initiation in Ethiopia, EDHS 2019

The spatial distribution of delayed breastfeeding initiation in Ethiopia is clustered with Global Moran’s I value of 0.17, *P*– value < 0.001 and Z-score 5.14. This finding revealed that delayed breastfeeding initiation in Ethiopia has a spatial dependency. Furthermore, the possibility of this clustered pattern to be because of random chance is less than 1%. (Fig. 2).

**Fig. 3:**
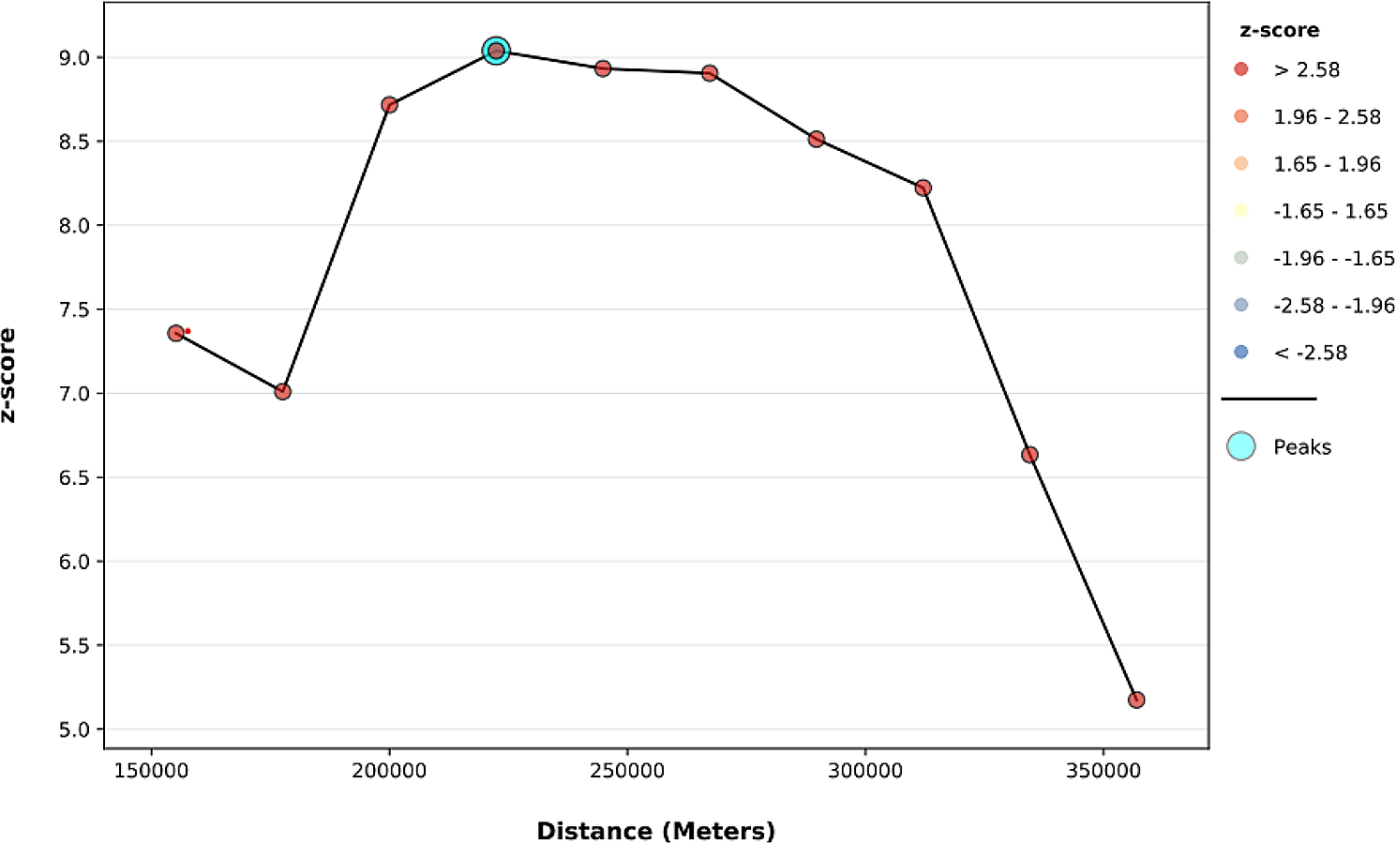
Incremental autocorrelation analysis of delayed breastfeeding initiation in Ethiopia, EDHS 2019

The line graph displays Incremental autocorrelation with distance to show the maximum and the minimum band. Thus, the minimum distance at the beginning was 155193.00 m (Z-score = 7.36, *P*– value < 0.001), whereas the first maximum peak was 222441.93 m (Z-score = 9.038, *P* - value < 0.001). (Fig. 3).

### Hot spot analysis

**Fig. 4:**
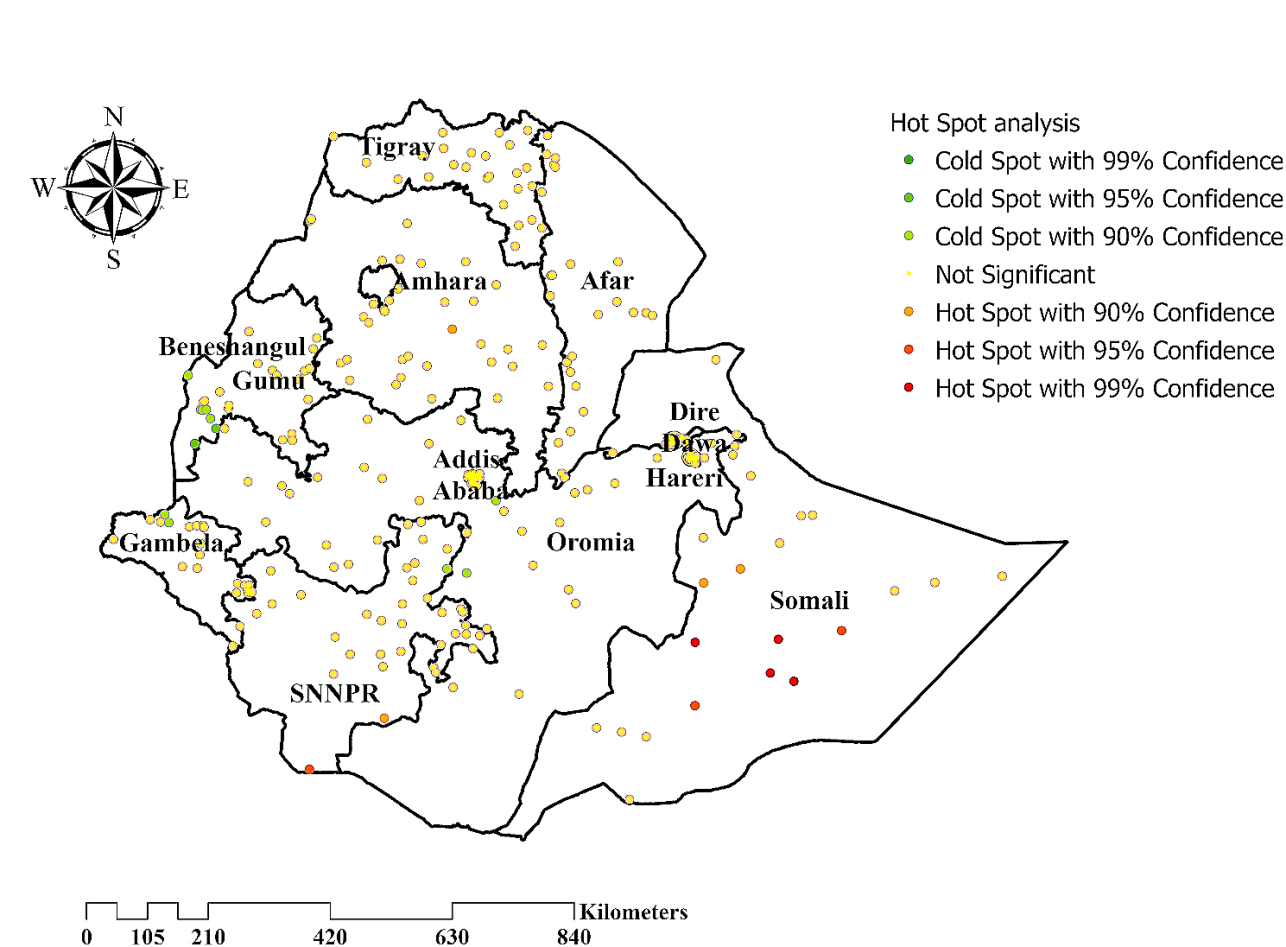
Hot spots analysis of delayed breastfeeding initiation in Ethiopia, EDHS 2019

Hot spot analysis was computed to detect areas with high and low values of delayed breastfeeding initiation. Thus, significant clustering of delayed breastfeeding initiation was mainly found in the Somali region. (Fig. 4).

### Spatial interpolation

**Fig. 5:**
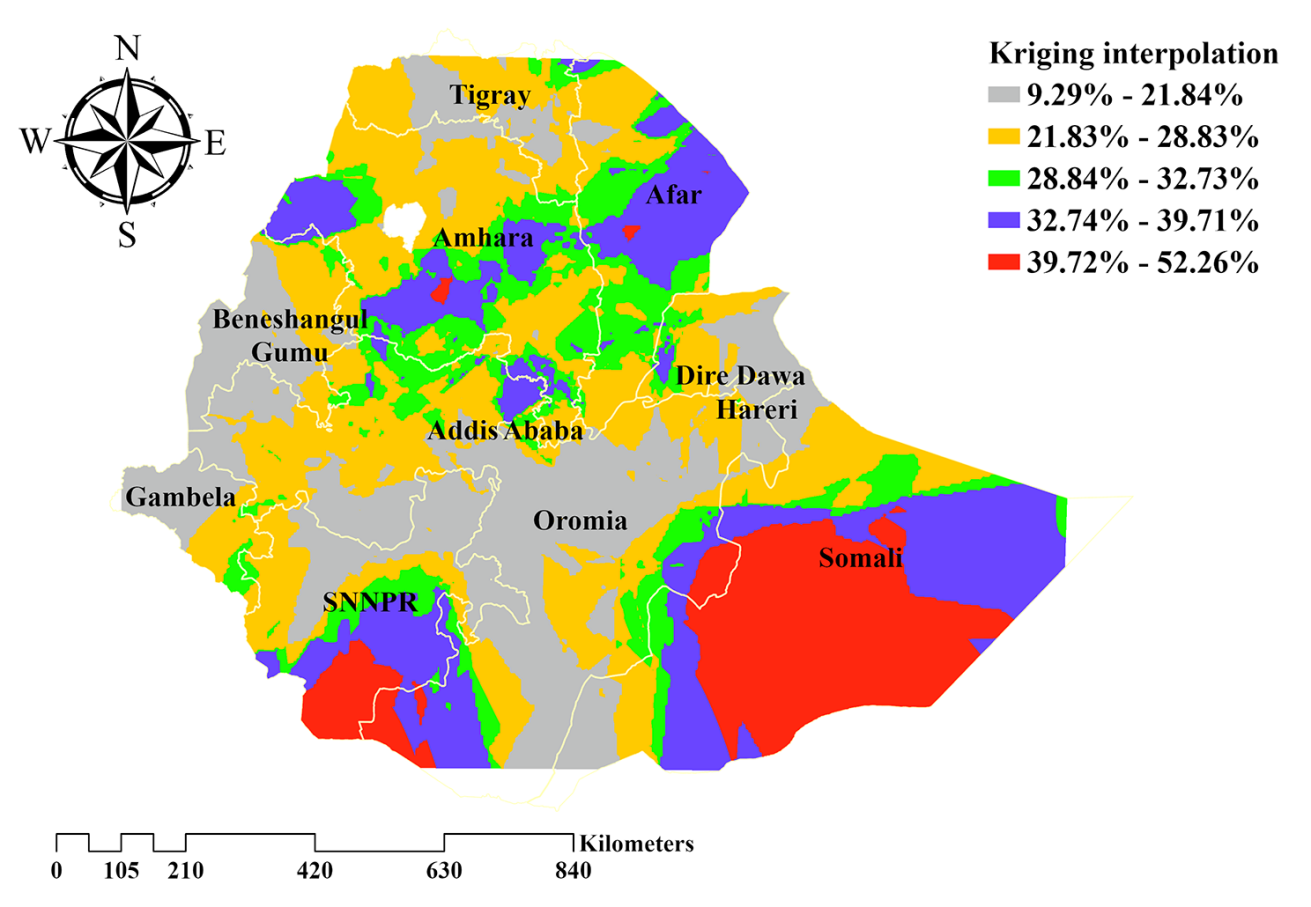
Kriging interpolation of delayed breastfeeding initiation in Ethiopia, EDHS 2019

Kriging interpolation technique was conducted to predict the distribution of delayed breastfeeding initiation in Ethiopia. Thus, the highest predicted prevalence of delayed breastfeeding initiation is observed in Ethiopia’s southern and eastern parts (Somali region). Whereas low prediction of delayed breastfeeding initiation is found in Western, Central and Northern Ethiopia. (Fig. 5).

### Satscan analysis

**Fig. 6:**
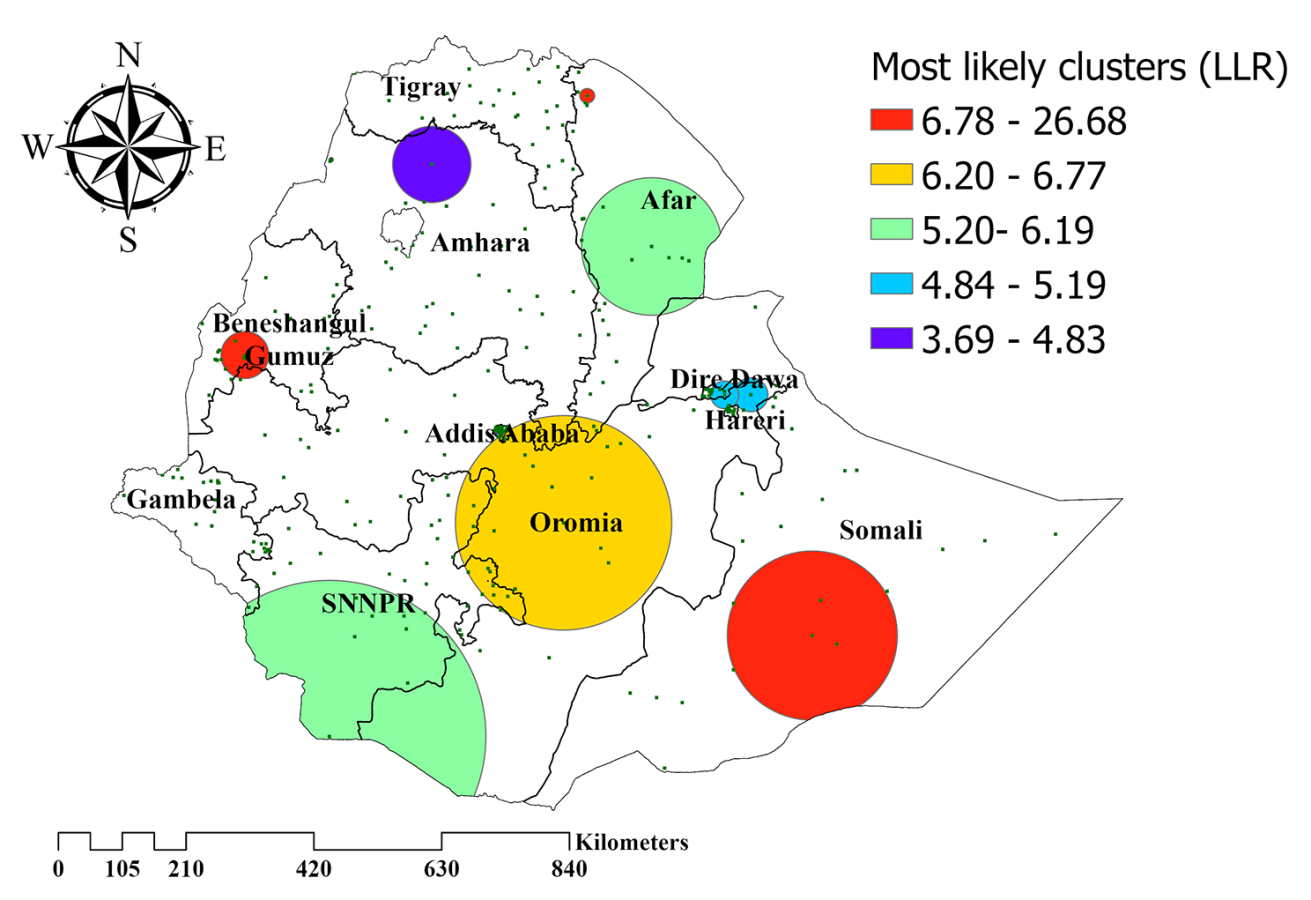
Spatial clustering of high and low rate of delayed breastfeeding initiation in Ethiopia, EDHS 2019

Purely spatial analysis using the Bernoulli model was done to identify clusters with high or low rates. The primary significant cluster was identified in Eastern Ethiopia (Somali region) at 5.984681 N, 43.361253 E geographic location (radius of 138.76 km), with a Relative risk of 3.14 (*P*– value ≤ 0.001) and Log likelihood ratio (LLR) of 26.68. Thus, children living in this region were more than three times delayed in breastfeeding initiation. The prevalence of delayed breastfeeding initiation was higher with the circle hole than the outside. (Fig. 6).

### Multicollinearity test

Variance inflation factor and tolerance value were used to check the exitances of multicollinearity between variables. A VIF above 4 or tolerance below 0.25 indicated that multicollinearity might exist. In this study the maximum VIF was 2.04 with mean VIF of 1.55 and the minimum tolerance value is 0.49. Thus, there is no multicollinearity between covariates. (Supplementary file 1).

### Multilevel analysis

#### Model comparison and random effect

The value of ICC in the null model shows that 17.5% of the difference in delayed breastfeeding initiation is as a result of cluster (enumeration area) difference. Furthermore, the MOR value in the null model revealed that 2.22 times the odds of difference in delayed breastfeeding initiation between study subjects is attributed to differences in clusters. The PCV in the final model explained that 13.5% variation in delayed breastfeeding initiation is because of individual and community-related factors. DIC and Log-likelihood were computed for model comparison. A model with a small DIC and high Log-likelihood was declared the best-fitted model. Thus, model 4 was the best-fitted model (DIC = 2213.34, Log-likelihood = -1095.01). (Table 4 ).

**Table 4:** Result of model comparison and random effect to determine variation of delayed breastfeeding initiation between clusters in Ethiopia, EDHS 2019

Parameter	Model 1	Model 2	Model 3	Model 4
ICC	17.5%	15.8%	17.1%	15.6%
MOR	2.22 (1.88, 2.73)	2.11 (1.78, 2.63	2.19 (1.86, 2.69)	2.10 (1.77, 2.61)
PCV	Ref	11.78%	3.12%	13.49%
Model comparison				
DIC	2289.46	2215.01	2289.95	2213.34
Log-likelihood	-1143.7548	-1096.1652	-1140.86	-1095.01

ICC, Intracluster correlatio; MOR, Median odds ratio; PCV, Proportional change in variance; DIC, deviation information criterion

### Determinants of delayed breastfeeding initiation

The result of multivariable multilevel mixed-effect logistic regression analysis (Model 4) revealed that the age of the mother, ANC visit, place of delivery and mode of delivery were found to be determinants of delayed breastfeeding initiation. The odds of delayed breastfeeding initiation among mothers with age groups of 15–24 and 25–34 were 1.66 (AOR 1.66; 95% CI 1.06, 2.62) and 1.57 (AOR 1.57; 95% CI 1.09, 2.25) times respectively high as compared to 35–49 year mother age group. Mothers who had no ANC visit were 45% more likely to delay breastfeeding initiation than the reverse group (AOR 1.45; 95% CI 1.04, 2.02). Home delivery increases the odds of delayed initiation of breastfeeding by 53% than giving birth in health institution (AOR 1.53; 95% CI 1.14, 2.06). Mothers who gave birth through cesarean section were 4.79 times (AOR 4.79; 95% CI 3.19, 7.21) more likely to delay initiation of breastfeeding as compared to spontaneous vaginal delivery. (Table 5).

**Table 5:** Multivariable multilevel logistic regression analysis of determinants of delayed breastfeeding initiation in Ethiopia, EDHS 2019

Variables	Model 2 AOR (95% CI)	Model 3 AOR (95% CI)	Model 4 AOR (95% CI)
Age of the mother			
15–24	1.74 (1.11, 2.74)**		1.66 (1.06, 2.62)**
25–34	1.59 (1.12, 2.28)**		1.57 (1.09, 2.25)**
35–49	1		1
Educational status			
No education	1		1
Primary education	1.02 (0.76, 1.35)		1.02 (0.77, 1.37)
Secondary education	0.95 (0.62, 1.46)		0.96 (0.64, 1.48)
Higher education	0.73 (0.44, 1.22)		0.75 (0.44, 1.26)
Marital status			
Married	1		1
Not married	0.76 (0.45, 1.25)		0.75 (0.45, 1.25)
Wealth index level			
Poor	1		1
Middle	0.98 (0.68, 1.43)		1.08 (0.74, 1.58)
Rich	0.97 (0.69, 135)		1.10 (0.78, 1.54)
Media access (television)			
No	1		1
Yes	0.99 (0.66, 1.49)		1.11 (0.71, 1.75)
Media access (radio)			
No	1		1
Yes	0.96 (0.75, 1.22)		0.97 (0.76, 1.24)
ANC visit			
No ANC visit	1.49 (1.08, 2.08)**		1.45 (1.04, 2.02)*
1–3 visit	1.11 (0.87, 1.42)		1.09 (0.85, 1.39)
4 and above visit	1		1
Place of delivery			
Health institution	1		1
Home	1.56 (1.15, 2.09)**		1.53 (1.14, 2.06)***
Mode of delivery			
SVD	1		1
Cesarean section	4.58 (3.04, 6.89)***		4.79 (3.19, 7.21)***
Child is Twin			
No	1		1
Yes	1.10 (0.37, 3.23)		1.08 (0.37, 3.18)
Parity			
1–2	1		1
3–4	0.82 (0.59, 1.12)		0.81 (0.59, 1.11)
5 and above	0.81 (0.49, 1.33)		0.79 (0.47, 1.30)
Birth order			
1	1		1
2–3	0.98 (0.77, 1.55)		0.91 (0.58, 1.47)
4 and above	0.99 (0.56, 1.89)		0.96 (0.57, 1.68)
Place of residence			
Urban		1	1
Rural		1.07 (0.79, 1.45)	1.10 (0.71, 1.73)
Community poverty level			
Lower		1	1
Higher		1.18 (0.98, 1.56)	1.17 (0.84, 1.62)
Region			
Metropolitan		1	1
Small peripheral		1.13 (082, 1.56)	1.08 (0.78, 1.49)
Large central		0.97 (0.69, 1.34)	0.84 (0.60, 1.16)

## Discussion

This study aimed to determine the spatial distribution and determinants of delayed breastfeeding initiation. The prevalence of delayed breastfeeding initiation is 26.4% (95% CI 24.4, 28.3). A high hotspot area was identified in the eastern part of Ethiopia. Maternal age, ANC visit, mode of delivery and place of delivery were found to be determinants of late initiation of breastfeeding practice.

In this study, the prevalence of late initiation of breastfeeding is higher than the study done in Ghana (18%) [29] and Sri Lanka (16.5%) [30]. On the contrary, the above finding is lower than the study done in the United Arab Emirates (37%) [31], Tanzania (49%) [32], Brazil (52.9%) [33], Nigeria (65.3%) [34] and Bangladesh (61.5%). This discrepancy might be attributed to variations in healthcare service utilization, community wealth level, culture, taboos towards the first breast milk (colostrum), religion and methodological differences between studies. Additionally, our findings concur with those of the previous study [35]. This consistency is likely attributable to the similarities in the characteristics of the populations studied and the settings in which the studies were conducted. Moreover, shared demographic characteristics, socioeconomic status, and lifestyle patterns may also contribute to this consistency.

This study found that maternal age is a determinant of late initiation of breastfeeding practice. Young mothers were more than one and a half times likely to delay breastfeeding initiation than older mothers. As the age of the mother increases, initiation of breastfeeding practice occurs earlier. This result is consistent with the study done in the United Arab Emirates [36]. The possible explanation might be that as the age of the mother increases, the likelihood of experiencing bad outcomes of late initiation of breastfeeding from the previous child rises. Besides, youth are more susceptible to unwanted pregnancies [37], and the newborn baby might not get all the necessary attention immediately after birth. Furthermore, young mothers might not have experience in the utilization of maternal health services, thereby, resulting in poor knowledge regarding the importance of early initiation of breastfeeding practice. Thus, incorporating health education sessions into youth and a more friendly service regarding the benefit of timely initiation of breastfeeding is crucial. Furthermore, public health interventions should prioritize providing tailored support and education to younger mothers to encourage early breastfeeding initiation.

Delayed breastfeeding initiation practice is significantly higher among mothers with no ANC visit than those who have received a visit. This result is supported by the study done in Uganda [38] and Namibia [18]. Mothers who had antenatal care visits might have heard about the significance of timely initiation of breastfeeding on child health and initiated breastfeeding earlier. Furthermore, the more ANC visits the women have, the more contact with a healthcare professional, thereby receiving health information. The higher prevalence of delayed breastfeeding initiation among mothers without ANC visits suggests that disparities in access to ANC services may contribute to this issue. Efforts should be made to improve access to antenatal care services, particularly in underserved communities.

Mothers who gave birth at home are more likely to have delayed initiation of breastfeeding practice than mothers who gave birth at health institutions. This finding was in line with the study done in Malawi [39] and Northern Uganda [40]. The possible reason for this disparity in delayed initiation of breastfeeding could be that a mother who gave birth at home might not exposed to health education and further instruction from healthcare professionals towards the health benefits of earlier initiation of breastfeeding. In Ethiopia, the prevalence of home delivery reaches up to sixty-six point 7% [41]. Reducing home delivery might significantly prevent adverse health outcomes in newborns who then experience delayed initiation of breastfeeding. Furthermore, community-based breastfeeding support programs can effectively provide education, counseling, and peer support to mothers who give birth at home, fostering a supportive environment for early breastfeeding initiation.

Delayed initiation of breastfeeding practice is significantly higher among children born through caesarean section. The study done in Bangladesh [17], United Arab Emirates [42], and South Sudan [11] also reports similar findings. Women who gave birth via caesarean section might not be conscious enough immediately after delivery to initiate breastfeeding as a result of the effects of anesthesia. Postoperative pain and discomfort following caesarean section can make it more difficult for mothers to initiate breastfeeding early. WHO implementation guidelines for early initiation of breastfeeding after a caesarean section birth assert that; when the woman is having difficulty of initiating breastfeeding due to any medical procedure, the child must be put on the breast immediately when she is conscious [43]. Therefore, any effort to reduce the CS rate enormously impacts improving breastfeeding initiation practice. Moreover, implementing baby-friendly hospital initiatives that foster practices conducive to early breastfeeding initiation, such as skin-to-skin contact, rooming-in, and lactation support, could also play a significant role.

This study uses nationally representative data. Thus, it can be generalizable at a country level and have better statistical power. Additionally, the study uses SaTScan and spatial distribution analysis, which is vital to show location-specific information regarding the problem. Due to the secondary nature of the data, the study’s ability to delve into the deeper causes of delayed breastfeeding initiation is constrained. The data may not capture all the relevant factors that contribute to delayed breastfeeding practices. The cross-sectional nature of the data restricts the ability to establish a causal link between the variables examined and delayed breastfeeding initiation. The absence of longitudinal data prevents the study from tracking changes in breastfeeding practices over time and identifying potential risk factors for delayed initiation.

## Conclusions

In Ethiopia, significant spatial culturing of delayed initiation of breastfeeding practice was found. Significant hotspot was identified in the eastern part (Somali region) of Ethiopia. Despite WHO recommendations to initiate breastfeeding immediately as possible, more than a quarter of newborn child start breastfeeding late. Maternal age, antenatal care visit, mode of delivery and place of delivery were significant determinants of delayed initiation of breastfeeding practice. Thus, programs encouraging health institution-based births and increasing ANC visits that promote early breastfeeding initiation are recommended. Guidelines and protocols should be developed regarding early initiation of breastfeeding after cesarean delivery. Particular emphasis is needed for young mothers. Including the benefit of timely initiation of breastfeeding into a youth-friendly service education program is very important. Furthermore, it is highly recommended that program planners develop effective interventions in high-hotspot areas.

### Electronic supplementary material

Below is the link to the electronic supplementary material.

Supplementary Material 1

## References

[1] World Health Organization. Fact sheets. Infant and young child feeding 2018 [Available from: https://www.who.int/news-room/fact-sheets/detail/infant-and-young-child-feeding.

[2] UNICEF W (2018). Capture the moment–early initiation of breastfeeding: the best start for every newborn.

[3] Raihana S, Alam A, Chad N, Huda TM, Dibley MJ (2021). Delayed initiation of Breastfeeding and Role of Mode and Place of Childbirth: evidence from health surveys in 58 low- and Middle- Income Countries (2012–2017). Int J Environ Res Public Health.

[4] Alebel A, Dejenu G, Mullu G, Abebe N, Gualu T, Eshetie S (2017). Timely initiation of breastfeeding and its association with birth place in Ethiopia: a systematic review and meta-analysis. Int Breastfeed J.

[5] Oakley L, Benova L, Macleod D, Lynch CA, Campbell OMR (2018). Early breastfeeding practices: descriptive analysis of recent demographic and health surveys. Matern Child Nutr.

[6] Al Sabati SY, Mousa O (2019). Effect of early initiation of breastfeeding on the uterine consistency and the amount of vaginal blood loss during early postpartum period. Nurs Prim Care.

[7] Marriott BP, White A, Hadden L, Davies JC, Wallingford JC (2012). World Health Organization (WHO) infant and young child feeding indicators: associations with growth measures in 14 low-income countries. Matern Child Nutr.

[8] Khan J, Vesel L, Bahl R, Martines JC (2015). Timing of breastfeeding initiation and exclusivity of breastfeeding during the first month of life: effects on neonatal mortality and morbidity–a systematic review and meta-analysis. Matern Child Health J.

[9] Teshale AB, Tesema GA (2021). Timely initiation of breastfeeding and associated factors among mothers having children less than two years of age in sub-saharan Africa: a multilevel analysis using recent demographic and health surveys data. PLoS ONE.

[10] Darboe ML, Jeyakumar A, Mansour SMA, Valawalkar S (2023). Determinants of early initiation of breastfeeding in the Gambia: a population-based study using the 2019–2020 demographic and health survey data. Int Breastfeed J.

[11] Bruno Tongun J, Sebit MB, Mukunya D, Ndeezi G, Nankabirwa V, Tylleskar T (2018). Factors associated with delayed initiation of breastfeeding: a cross-sectional study in South Sudan. Int Breastfeed J.

[12] Yohannes E, Tesfaye T (2020). Timely initiation of breastfeeding and associated factors among mothers who have infants less than six months of age in Gunchire town, southern Ethiopia 2019. Clin J Obstet Gynecol.

[13] Senanayake P, O’Connor E, Ogbo FA (2019). National and rural-urban prevalence and determinants of early initiation of breastfeeding in India. BMC Public Health.

[14] Takahashi K, Ganchimeg T, Ota E, Vogel JP, Souza JP, Laopaiboon M (2017). Prevalence of early initiation of breastfeeding and determinants of delayed initiation of breastfeeding: secondary analysis of the WHO Global Survey. Sci Rep.

[15] Seidu AA, Ameyaw EK, Ahinkorah BO, Bonsu F (2020). Determinants of early initiation of breastfeeding in Ghana: a population-based cross-sectional study using the 2014 demographic and Health Survey data. BMC Pregnancy Childbirth.

[16] Kitsantas P, Pawloski LR (2010). Maternal obesity, health status during pregnancy, and breastfeeding initiation and duration. J Matern Fetal Neonatal Med.

[17] Raihana S, Alam A, Huda TM, Dibley MJ (2021). Factors associated with delayed initiation of breastfeeding in health facilities: secondary analysis of Bangladesh demographic and health survey 2014. Int Breastfeed J.

[18] Ahmed KY, Page A, Arora A, Ogbo FA (2019). Trends and determinants of early initiation of breastfeeding and exclusive breastfeeding in Ethiopia from 2000 to 2016. Int Breastfeed J.

[19] Hailegebreal S, Haile Y, Seboka BT, Enyew EB, Shibiru T, Mekonnen ZA (2022). Modeling spatial determinants of initiation of breastfeeding in Ethiopia: a geographically weighted regression analysis. PLoS ONE.

[20] Horii N, Guyon AB, Quinn VJ (2011). Determinants of delayed initiation of breastfeeding in rural Ethiopia: programmatic implications. Food Nutr Bull.

[21] Colglazier W (2015). Sustainable development agenda: 2030. Science.

[22] Ethiopian Federal Ministry of Health. Health sector transformation plan (HSTP II): 2020/21-2024/25 (2013 EFY– 2017 EFY). 2021.

[23] Ethiopia Population.– 2021 Data– 2022 Forecast– 1960–2020 Historical - Chart - News 2022 [Available from: https://tradingeconomics.com/ethiopia/population.

[24] Ethiopian Public Health Institute (EPHI) and ICF (2019). Ethiopia Mini Demographic and Health Survey. Final report.

[25] World Health Organization. Fact sheets. Infant and young child feeding. 2021.

[26] Bosker R, Snijders TA. Multilevel analysis: an introduction to basic and advanced multilevel modeling. Multilevel Anal. 2011.

[27] Kitaw TA, Haile RN (2022). Time to first antenatal care booking and its determinants among pregnant women in Ethiopia: survival analysis of recent evidence from EDHS 2019. BMC Pregnancy Childbirth.

[28] Rabe-Hesketh S, Skrondal A, editors. Understanding variability in multilevel models for categorical responses. Proceedings of the AERA Annual Meeting, Vancouver, BC, Canada; 2012.

[29] Dubik SD, Amegah KE (2021). Prevalence and determinants of early initiation of breastfeeding (EIBF) and prelacteal feeding in Northern Ghana: a cross-sectional survey. PLoS ONE.

[30] Senarath U, Siriwardena I, Godakandage SS, Jayawickrama H, Fernando DN, Dibley MJ (2012). Determinants of breastfeeding practices: an analysis of the Sri Lanka Demographic and Health Survey 2006–2007. Matern Child Nutr.

[31] Taha Z, Al Dhaheri AI, Wikkeling-Scott L, Ali Hassan A, Papandreou D. Determinants of delayed initiation of breastfeeding: a cross-sectional Multicenter Study in Abu Dhabi, the United Arab Emirates. Int J Environ Res Public Health. 2022;19(15). 10.3390/ijerph19159048. 10.3390/ijerph19159048 PMC9331463 35897420

[32] Exavery A, Kanté AM, Hingora A, Phillips JF (2015). Determinants of early initiation of breastfeeding in rural Tanzania. Int Breastfeed J.

[33] Vieira TO, Vieira GO, Giugliani ER, Mendes CM, Martins CC, Silva LR (2010). Determinants of breastfeeding initiation within the first hour of life in a Brazilian population: cross-sectional study. BMC Public Health.

[34] Berde AS, Yalcin SS (2016). Determinants of early initiation of breastfeeding in Nigeria: a population-based study using the 2013 demograhic and health survey data. BMC Pregnancy Childbirth.

[35] John JR, Mistry SK, Kebede G, Manohar N, Arora A (2019). Determinants of early initiation of breastfeeding in Ethiopia: a population-based study using the 2016 demographic and health survey data. BMC Pregnancy Childbirth.

[36] Radwan H (2013). Patterns and determinants of breastfeeding and complementary feeding practices of Emirati Mothers in the United Arab Emirates. BMC Public Health.

[37] Mamboleo N. Unwanted pregnancy and induced abortion among female youths: a case study of Temeke district. Muhimbili University of Health and Allied Sciences; 2012.

[38] Kusasira L, Mukunya D, Obakiro S, Kenedy K, Rebecca N, Ssenyonga L (2023). Prevalence and predictors of delayed initiation of breastfeeding among postnatal women at a tertiary hospital in Eastern Uganda: a cross-sectional study. Arch Public Health.

[39] Kazembe LN (2008). Spatial modelling of initiation and duration of breastfeeding: analysis of breastfeeding behaviour in Malawi - I. World Health Popul.

[40] Mukunya D, Tumwine JK, Nankabirwa V, Ndeezi G, Odongo I, Tumuhamye J (2017). Factors associated with delayed initiation of breastfeeding: a survey in Northern Uganda. Glob Health Action.

[41] Ayenew AA, Nigussie AA, Zewdu BF (2021). Childbirth at home and associated factors in Ethiopia: a systematic review and meta-analysis. Arch Public Health.

[42] Taha Z, Ali Hassan A, Wikkeling-Scott L, Papandreou D (2021). Factors Associated with delayed initiation and Cessation of Breastfeeding among Working Mothers in Abu Dhabi, the United Arab Emirates. Int J Womens Health.

[43] World Health Organization. Implementation guidance: protecting, promoting and supporting breastfeeding in facilities providing maternity and newborn services: the revised baby-friendly hospital initiative. 2018. 29565522

