# Supplementary material for: Spatial variation and determinants of delayed breastfeeding initiation in Ethiopia: spatial and multilevel analysis of recent evidence from EDHS 2019
Source: Int Breastfeed J. 2024 Feb 7;19:10. doi: 10.1186/s13006-024-00616-1 (PMC10851470; doi:10.1186/s13006-024-00616-1)
Supplement: Supplementary file 1 — Supplementary Material 1 [file 13006_2024_616_MOESM1_ESM.pdf]

Multicollinearity test

Variance inflation factor and tolerance value were used to check the exitances of multicollinearity between variables. A VIF above 4 or tolerance below 0.25 indicated that multicollinearity might exist. In this study the maximum VIF was 2.04 with mean VIF of 1.55 and the minimum tolerance value is 0.49. Thus, there is no multicollinearity between covariates. *(Table 1).*

Table 1: VIF and tolerance test to check the existence of multicollinearity between covariates.

Variables	*VIF*	*1/VIF*
Birth order	2.04	0.490196078
Parity	2.01	0.497512438
Wealth index level	1.94	0.515463918
Place of residence	1.90	0.526315789
Media access (television)	1.85	0.540540541
Age of the mother	1.84	0.543478261
Community poverty level	1.77	0.564971751
Place of delivery	1.68	0.595238095
Educational status	1.63	0.613496933
ANC visit	1.47	0.680272109
Region	1.29	0.775193798
Media access (radio)	1.14	0.877192982
Mode of delivery	1.13	0.884955752
Marital status	1.03	0.970873786
Child is Twin	1.03	0.970873786
Mean VIF	1.55	
